# Lithium-Rich Rock Salt Type Sulfides-Selenides (Li_2_TiSe_x_S_3−x_): High Energy Cathode Materials for Lithium-Ion Batteries

**DOI:** 10.3390/ma15093037

**Published:** 2022-04-22

**Authors:** Yagmur Celasun, Jean-François Colin, Sébastien Martinet, Anass Benayad, David Peralta

**Affiliations:** CEA, LITEN, University Grenoble Alpes, F-38054 Grenoble, France; yagmurcelasun@hotmail.com (Y.C.); jean-francois.colin@cea.fr (J.-F.C.); sebastien.martinet@cea.fr (S.M.); anass.benayad@cea.fr (A.B.)

**Keywords:** high-energy materials, sulfides, anionic redox, wet mechanochemistry, selenium substitution, cyclic voltammetry

## Abstract

Lithium-rich disordered rocksalt Li_2_TiS_3_ offers large discharge capacities (>350 mAh·g^−1^) and can be considered a promising cathode material for high-energy lithium-ion battery applications. However, the quick fading of the specific capacity results in a poor cycle life of the system, especially when liquid electrolyte-based batteries are used. Our efforts to solve the cycling stability problem resulted in the discovery of new high-energy selenium-substituted materials (Li_2_TiSe_x_S_3−x_), which were prepared using a wet mechanochemistry process. X-ray diffraction analysis confirmed that all compositions were obtained in cation-disordered rocksalt phase and that the lattice parameters were expanded by selenium substitution. Substituted materials delivered large reversible capacities, with smaller average potentials, and their cycling stability was superior compared to Li_2_TiS_3_ upon cycling at a rate of C/10 between 3.0–1.6 V vs. Li^+^/Li.

## 1. Introduction

The rapid growth in electric vehicle market requires high performance, safe, and low-cost battery packs that should enable driving ranges exceeding 500 km. Current Li-Ion battery positive electrode materials can realize this objective by providing specific energies that exceed 250 Wh·kg^−1^ at cell level [1,2,3,4]. These materials still contain a small percentage of cobalt, and the concerns related to this critical element have increased, due to its price and availability. Recent research efforts have focused on the discovery of Co-poor or even Co-less positive electrode materials and the development of alternative high-energy materials, such as lithium sulfide and cation-disordered rocksalts [5,6,7,8,9,10,11,12,13,14,15,16,17,18,19,20]. Among them, cation-disordered rocksalts have recently received great interest, as a new generation of positive electrode materials for lithium ion batteries [16,17,21,22]. For instance, disordered rocksalt sulfides deliver high specific capacities (>400 mAh·g^−1^) at relatively low operating potentials (~2.2 V vs. Li^+^/Li), and their energy density is competitive with conventional layered materials [19]. This low operating potential may be considered an advantage of sulfide-type batteries, since they do not experience the serious electrolyte degradation problems occurring at higher voltages [23]. In addition, these materials allow the exchange of more than one Li per metal cation (multi-electrons redox reactions); thus, greater capacities could be produced [19,24,25]. Such a property was already noticed and reported in earlier studies, as TiS_3_ was able to host three Li^+^ ions during discharge [26,27]. However, only one lithium ion was reversibly intercalated [26]. 

More recently, new studies have paved the way for the discovery of novel Li-rich disordered rocksalt sulfides. Sakuda et al. highlighted that Li_2_TiS_3_ and Li_3_NbS_4_ materials with a disordered rocksalt cubic structure can provide capacities above 400 mAh·g^−1^ upon cycling between 3–1.5 V vs. Li^+^/Li [19,24]. Despite their promising reversible capacities, the retention performances of Li_2_TiS_3_ and Li_3_NbS_4_ were rather poor in conventional cells; on the contrary, superior retention performances were achieved in solid-state cells [16]. For industrial applications, materials should have both a long shelf life and a high capacity. The retention performance of Li_2_TiS_3_ is a major bottleneck, and new approaches are necessary to improve this. Partial substitution mechanisms may be promising solutions for this issue. For instance, sulfur anions could be replaced with an alternative anion. Studies show that selenium substitution can improve the cycling stability of Li_2_S cells [28,29]; therefore, such results motivated us to substitute sulfur with selenium in Li_2_TiS_3_. Thanks to an optimized wet milling process, two new lithium-rich rock salt type sulfides–selenides compounds (Li_2_TiSe_x_S_3−x_) are reported in this study, for the first time. Structural investigations, as well as electrochemical tests have been performed, to compare the mechanism of lithium insertion/extraction between these new compounds and the previously published Li_2_TiS_3_ cathode material [19].

## 2. Materials and Methods

### 2.1. Wet Mechanochemical Synthesis of Li_2_TiSe_x_S_3−x_

According to the synthesis procedure; 1.5 g of precursors composed of lithium sulfide (Li_2_S, Sigma Aldrich, St. Louis, MO, USA, 98%), titanium powder (Alfa Aesar, Havrier, MA, USA, 99.98%), sulfur powder (Sigma Aldrich, 99.99%), and selenium powder (Alfa Aesar, 99.9%) precursors were placed into a 50-mL zirconia jar that contained 285 zirconia balls (5 mm in diameter). Then, an appropriate amount of anhydrous hexane (99.9%, Sigma Aldrich) was added, until covering the entire ball surface. Afterwards, the jar was closed tightly and transferred from an argon-filled glovebox to the planetary ball milling machine (Retsch PM 100). Milling was operated at 510 rpm for 20 hours. Powder was recovered in an argon-filled glovebox, to limit air contamination in the powders. Zirconia impurities due to high-energy milling were checked using inductively coupled plasma atomic emission spectroscopy measurements and were considered low (0.018 mole of ZrO_2_ for 1 mole of Li_2_TiS_3_).

### 2.2. Structural Characterizations

X-ray diffraction (XRD) analyses were carried out on a BRUKER AXS D8 diffractometer, using Cu Kα (λ = 1.5406 Å) anticathode, where the sample was scanned in the range of 8–80° using a step size of 0.02°. As the sulfide materials are air sensitive, all of the samples were prepared in a glovebox, and the sample surface was protected with Kapton^®^. A signal due to Kapton^®^ film could be detected at small 2θ angles (0–28°) in the diffraction patterns of the powders. 

Scanning electron microscopy (SEM) and energy-dispersive X-ray spectroscopy (EDX) were performed with a microscope, Zeiss brand MEB LEO 1530 Gemini. 

Chemical characterization by X-ray photoelectron spectroscopy (XPS) was carried out using a Versaprobe II ULVAC-PHI spectrometer. A monochromatic beam (X-ray source Al Kα 1486.6 eV) of 100 μm diameter and 25 W of power was focused on the surface of the samples. Survey spectra were measured over a spectral range of 0−1200 eV, to identify the elements present in the material using a pass energy of 117 eV, which corresponds to a resolution of 1.6 eV. High resolution spectral analyses were performed using a pass energy of 23 eV, which corresponds to a resolution of 0.5 eV. All XPS measurements were carried out in an ultrahigh vacuum chamber (7 × 10^−10^ mbar). All XPS spectra binding energies were corrected using the C1s line of alkyl groups in the C-C at 285.0 eV. Curve fitting and background subtraction were accomplished using Casa XPS software. The spectra curve fitting was performed using the pseudo-Voigt function, product of Gaussian (80%), and Lorentzian (20%) distributions.

### 2.3. Electrochemical Characterizations

Electrode preparation: In an Ar-filled glove box, positive electrodes were prepared using 80 wt.% of the active material, 10 wt.% Carbon SuperP (Timcal, Willebroek, Belgium), 10 wt.% of PVdF (Solvay, Brussels, Belgium). An adequate amount of NMP (Sigma Aldrich) was added, to form a uniform slurry, then it was coated on an aluminum foil (20 μm) using a 100 µm doctor blade and left to dry in the glovebox for three days. The electrodes were cut (14 mm diameter), pressed (10 t), weighed (loaded > 2 mg) in the glovebox, and dried in a Buchi^®^ oven at 60 °C for 12 h under vacuum. 

Electrochemical testing: Coin cells were prepared using 14-mm diameter positive electrodes, 16-mm diameter Li foil as a negative electrode, and 150 μL electrolyte containing 1 M LiPF_6_ in EC:PC:DMC, where EC: ethylene carbonate; PC: propylene carbonate; DMC: dimethyl carbonate, in a volumetric ratio of 1:1:3, respectively. A propylene separator (Celgard^®^ 2400, 16.5 mm diameter) and polyolefin separator (Viledon^®^, 16.5 mm diameter) were used in the coin cells. Three-coin cells were cycled at 22 °C, at a rate of C/10 between 3 V and 1.5 V, using ARBIN cycling instrumentation.

## 3. Results

**Figure 1:**
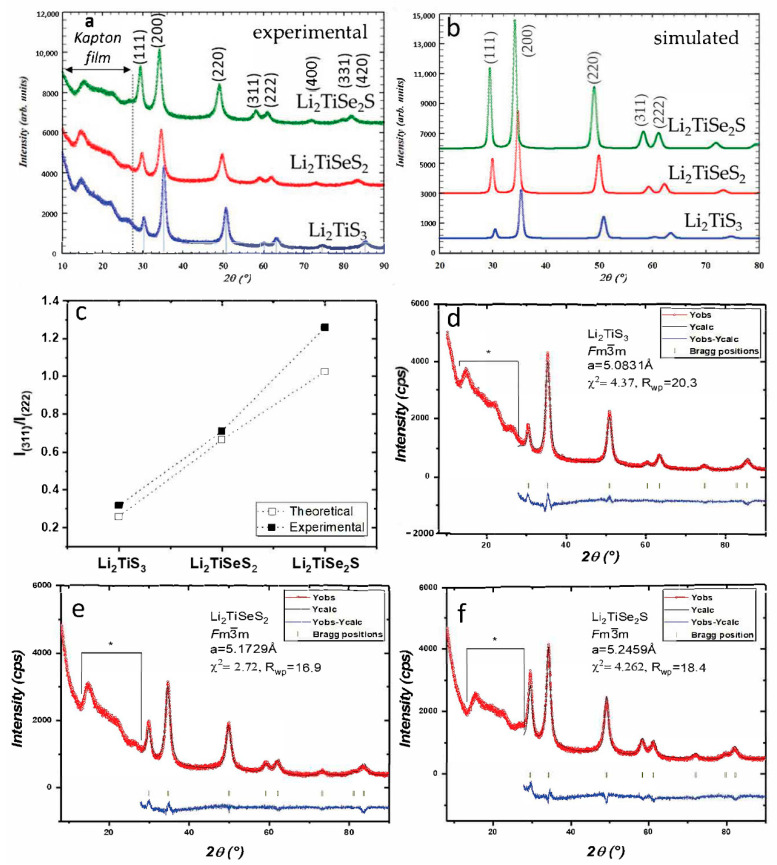
(**a**)/XRD patterns of Li_2_TiSe_x_S_3−x_ (0 ≤ x ≤ 2) materials—(**b**)/Simulated XRD patterns of Li_2_TiSe_x_S_3−x_ powders and (**c**)/experimental and simulated peak intensity ratio of (311) and (222) planes, I_(311)_/I_(222)_ for Li_2_TiS_3_, Li_2_TiSeS_2_, and Li_2_TiSe_2_S powders—(**d**–**f**)/XRD pattern of Li_2_TiSe_x_S_3−x_ (0 ≤ x ≤ 2) Rietveld refinement in the Fullprof program. Brackets with asterisk indicate the zone where the protective Kapton^®^ foil signal prevented the refinement from being carried out.

To prepare selenium substituted Li_2_TiS_3_ compositions (Li_2_TiSe_x_S_3−x_), we developed a flexible and easy process utilizing ball-milling, in which precursors are used in their neutral form, as neither Li_2_Se nor TiSe_2_ precursors were commercially available [30]. Our synthesis process does not require applying multiple steps to prepare the ceramic powders [28]. The powder diffraction patterns of the three resulting powders (Li_2_TiSe_x_S_3−x_) are presented in Figure 1a. 

With our original synthesis process (wet mechanochemistry), Li_2_TiS_3_ was successfully prepared as a disordered rocksalt phase. Selenium substituted samples showed a similar peak profile as Li_2_TiS_3_, indicating that Li_2_TiSe_x_S_3−x_ compositions were also prepared in disordered rocksalt phase. 

**Figure 2:**
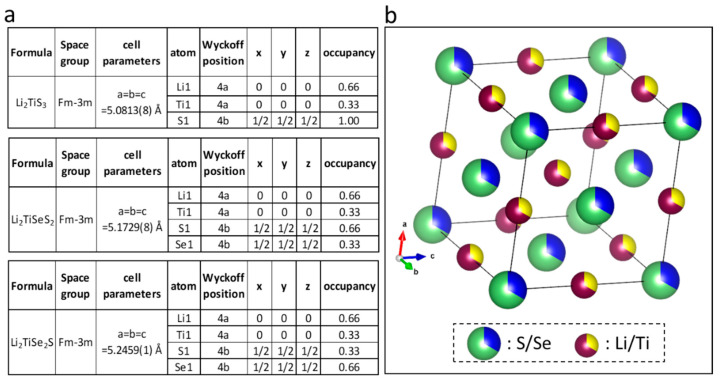
(**a**) Refined parameters of Li_2_TiSe_x_S_3−x_ (0 ≤ x ≤ 2) materials and (**b**) Li_2_TiSeS_2_ structure.

At first observation, the diffraction peaks of Li_2_TiSeS_2_ and Li_2_TiSe_2_S patterns shifted to small 2θ angles, indicating that the lattice parameters expanded with selenium substitution. This behavior was expected, as Se^2−^ is bigger than S^2−^. The lattice parameters of Li_2_TiSe_x_S_3−x_ powders were refined with Rietveld refinement in the Fullprof program [31]. All of the peak profiles are illustrated in Figure 1d–f, and the refined parameters and the resulting structure is shown in Figure 2. The lattice parameters of Li_2_TiS_3_ (5.0831(8) Å) were found to be slightly bigger than those previously reported (5.05 Å) [16]. Second, lattice parameters of Li_2_TiSeS_2_ and Li_2_TiSe_2_S were found (5.1729(8) Å) and (5.2459(1) Å), respectively.

In addition, the peak intensity ratios of Li_2_TiSeS_2_ and Li_2_TiSe_2_S differed from the peak intensity ratio of Li_2_TiS_3_ powder, as can be clearly seen on the intensity of the (311) and (222) reflections around 60°. We, thus, simulated the diffraction patterns of Li_2_TiSeS_2_ and Li_2_TiSe_2_S using the refined cell parameters and a similar structure as for Li_2_TiS_3_ (Figure 1b). The same evolution in peak intensity ratios was observed with selenium substitution, confirming the Se substitution. For the Rietveld refinements, owing to the poor crystallinity of the material and the presence of a broad signal coming from the Kapton^®^ protective film, it was decided to not refine the atomic occupancies and keep the model structures. However, without further refining, the agreement between the observed and calculated pattern was already quite good and confirmed that Se substitution had occurred in these samples.

**Figure 3:**
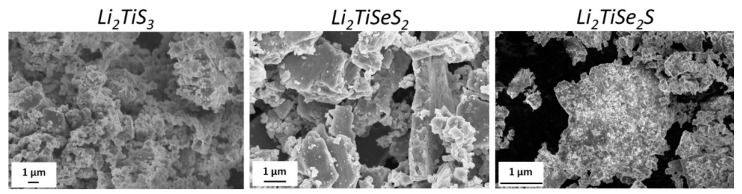
SEM images of Li_2_TiSe_x_S_3−x_ (0 ≤ x ≤ 2) materials.

The Figure 3 shows SEM images of Li_2_TiSe_x_S_3−x_ powders.

All powders were composed of agglomerates of primary nanoparticles, and, curiously, the Li_2_TiSeS_2_ agglomerates tended to have a plate-like morphology.

**Figure 4:**
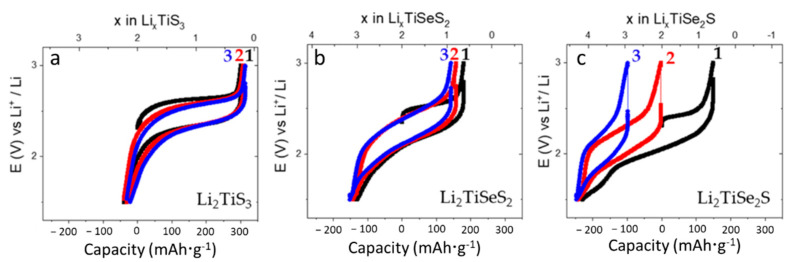
Galvanostatic curves of (**a**) Li_x_TiS_3_, (**b**) Li_x_TiSeS_2_, and (**c**) Li_x_TiSe_2_S cathode materials in half-cell at a rate of C/10 between 3 and 1.5 V vs. Li^+^/Li, the first three cycles were represented in the figures.

The electrochemical performances of Li_2_TiS_3_ and selenium-substituted samples (Li_2_TiSeS_2_ and Li_2_TiSe_2_S) were investigated and compared in half-cells (Figure 4).

The open circuit potential of Li_2_TiS_3_ was measured as 2.31 V at the beginning of the cycling. When the Li_2_TiS_3_ was charged with a C-rate of C/10 until 3 V, the 1.77 Li^+^ ions were extracted from Li_2_TiS_3_ and a capacity of 300 mAh·g^−1^ was delivered. During discharge, 2.0 Li^+^ ions were inserted into the structure, the composition changed into Li_2.23_TiS_3_, and a capacity of 339 mAh·g^−1^ was delivered. The average charge and discharge potentials were measured at 2.46 V and 2.23 V, respectively, and a summary of the results is shown in Table 1.

The Li_2_TiS_3_ material prepared using our original synthesis process delivered greater charge capacities, but slightly smaller discharge capacities, than the Li_2_TiS_3_ material previously reported [16]. Moreover, we observed that Li_2_TiS_3_ provided a greater discharge capacity at the initial cycle, but the charge–discharge curve was fully reversible at the end of the second and third cycles.

Charge: Li_2_Ti^4+^S_3_^2−^ → Li_0.23_Ti^4+^(S^2−^)_1.23_(S_2_^2−^)_0.885_ + 1.77 Li^+^ + 1.77 e^−^ (1)

Discharge: Li_0.23_Ti^4+^(S^2−^)_1.23_(S_2_^2−^)_0.885_ + 2 Li^+^ + 2 e^−^→ Li_2.23_(Ti^4+^)_x_(Ti^3+^)_1−x_(S^2−^)_3−y_(S_2_^2−^)_y_ (2)

with 2·(0.885 − y) + x = 2.

Such greater discharge capacity at the initial cycle was previously reported for both Li_2_TiS_3_ and other compositions (Li_3_NbS_4_ and Li_3_SnS_4_) [16,24,25]. We have to keep in mind that this phenomenon will require the addition of “lithium sacrificial salt” inside Li-ion cells if this material is used in front of a graphite anode instead of metal lithium. Another way to overcome this phenomenon could be to use the material in all solid-state batteries, with metal lithium as anode. In all cases, the origin of the extra capacity remains unknown, and further structural analyses are needed to explain it. 

We explain the possible redox mechanism in Li_2_TiS_3_ with a hypothesis whereby all the atoms are stabilized at their valence states (Ti^4+^ and S^2−^) in the pristine electrode, and there is no loss of sulfur atoms during the charge–discharge process. Based on this hypothesis, only sulfur redox should be active during charging, since Ti is already at its maximum valence of Ti^4+^. Therefore, charge capacity may have been produced by anionic (sulfur) redox, with some part of the S^2−^ atoms oxidized into S_2_^2^ (Equation (1)). Moreover, the discharge capacity could have been provided by either anionic redox (S^2−^/S_2_^2−^) or both anionic and cationic (Ti^4+^/Ti^3+^) redox processes. A reversible sulfur redox process was previously mentioned in the literature [32]. Reversible formation and dissociation of covalent S-S bonds in an Li_2_TiS_3_ electrode were detected in both pair distribution function analyses and ab initio molecular dynamics calculations, and this was later attributed to reversible sulfur redox processes.

Li_2_TiSeS_2_ and Li_2_TiSe_2_S cells were also tested with the same cycling schedule applied to Li_2_TiS_3_ cells, and 179 mAh·g^−1^ charge and 310 mAh·g^−1^ discharge capacities were delivered between 3 and 1.5 V vs. Li^+^/Li. The average charge and discharge potentials were measured to be 2.34 V and 2.06 V, respectively, indicating that the average potentials are reduced by selenium substitution. At the end of the first cycle, we again detected a large discharge capacity. More than 2.0 Li^+^ ions were inserted into the structure, and the composition changed into Li_3.08_TiSeS_2_, which can be regarded as the average (theoretical) composition. Such capacity was even greater than the theoretical capacity of Li_2_TiSeS_2_ cells (261 mAh·g^−1^ based on two electron exchange processes). At the subsequent cycles, a reversible cycling curve was observed. If we keep the same hypothesis that we previously used to explain the redox process of Li_2_TiS_3_ cells, the charge capacity should be produced by either sulfur (S^2^^−^/S_2_^2^^−^) or selenium (Se^2^^−^/Se_2_^2^^−^) redox processes, which is active between 3 and 1.5 V [29,30]. Here, again, the discharge capacity should be provided by either anionic (S^2^^−^/S_2_^2^^−^ or Se^2^^−^/Se_2_^2^^−^) or both anionic and cationic (Ti^4+^/Ti^3+^) redox processes.

Li_2_TiSe_2_S cells delivered charge and discharge capacities of 149 mAh·g^−1^ and 379 mAh·g^−1^, respectively. Much lower average charge and discharge potentials (2.24 V and 1.98 V) were detected. At the end of the initial discharge, 3.44 Li^+^ ions were inserted into the cubic rocksalt structure of Li_2_TiSe_2_S, and the theoretical composition became equivalent to Li_4.17_TiSe_2_S. We again detected extra discharge capacity, and this was even greater than the theoretical capacity of Li_2_TiSe_2_S (213 mAh·g^−1^ based on two electron exchange processes). Now, we cannot explain more than a three Li^+^ uptake with the same hypothesis; a combination of anionic and Ti^3+^/Ti^4+^ redox processes. In the cycling curve of Li_2_TiSe_2_S cells, we observed that the second and third cycles were reversible; however, smaller charge and discharge capacities, as well as a rapid capacity fading, were detected. 

**Figure 5:**
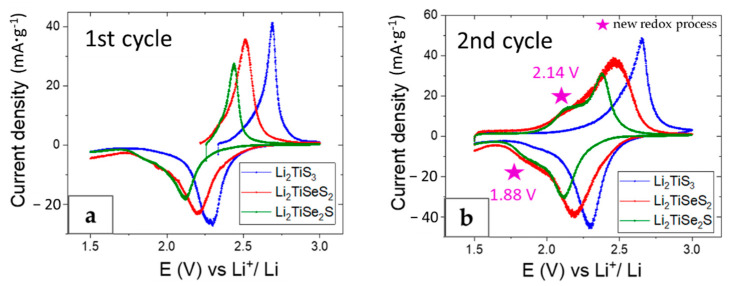
Redox potentials comparison of Li_2_TiS_3_, Li_2_TiSeS_2_, and Li_2_TiSe_2_S electrodes at the first (5 µV·s^−1^) (**a**), and second (10 µV·s^−1^) (**b**) cycles between 3 and 1.5 V vs. Li^+^/Li.

To describe the possible redox processes taking place in Li_2_TiSe_x_S_3−x_ cells, we conducted cyclic voltammetry measurements (Figure 5). 

Li_2_TiS_3_ cells showed one oxidative and one reductive peak that resulted from sulfur redox reaction (S^2−^/S_2_^2−^ and S_2_^2−^/S^2−^) at 2.69 V and 2.28 V. Moreover, 2.51 V charge and 2.20 V discharge potentials were detected in Li_2_TiSeS_2_ cells, and 2.43 V charge and 2.12 V discharge potentials were detected in Li_2_TiSe_2_S cells, in accordance with previous results showing a reduced working potential for Se-substituted materials. During discharge, the apparition of a second reduction peak could be observed as a shoulder of the main peak, only for the Se-substituted materials. This peak is reversible and can be observed in the second cycle of Li_2_TiSe_x_S_3−x_ cells. Therefore, we can assume that the substitution of S by Se in the Li_2_TiSe_x_S_3−x_ materials leads to the activation of a new redox process, which is the origin of the extra capacity.

**Figure 6:**
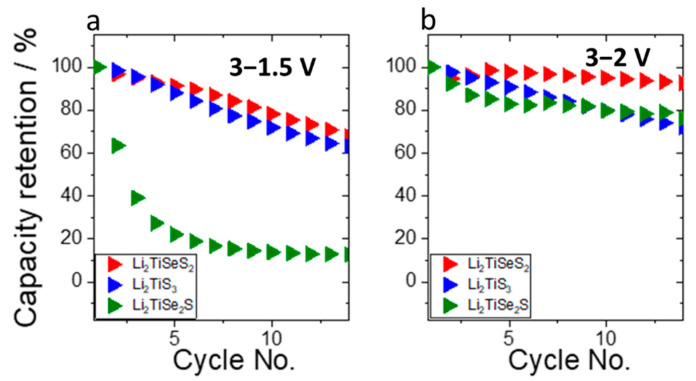
Capacity retention (%) of Li_2_TiS_3_, Li_2_TiSeS_2_, and Li_2_TiSe_2_S cells cycled at a rate of C/10 in different cycling windows: (**a**) 3–1.5 V and (**b**) 3–2.0 V vs. Li^+^/Li.

To investigate the effect of this supplementary process on the cycling stability of the materials, we carried out galvanostatic cyclings with two different low voltage cut-offs: one with a cut-off at 1.5 V, allowing the cycling on the supplementary process; and one at 2 V, to avoid the major part of this process. The results presented in Figure 6 show that the two substituted samples clearly benefited from the reduced voltage window. The improvement is more important for Li_2_TiSe_2_S, for which the low potential process represents a bigger part of the redox processes, with the capacity retention at the 14th cycle improving from 12% to 76%. It is, thus, reasonable to assume that the supplementary process is a major factor in the performance degradation of these materials. The stability of the non-substituted material was not significantly modified; this was expected, as no major redox process occurs in the 1.5–2 V region for this material. 

**Figure 7:**
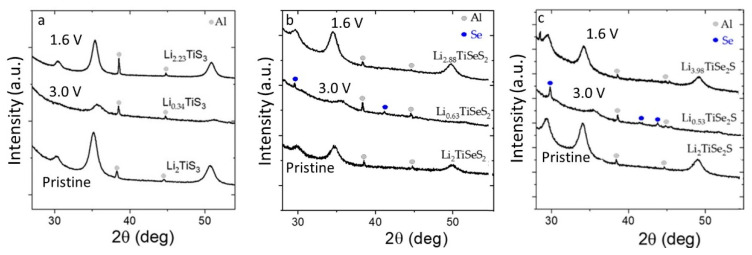
Ex situ X-ray diffractograms of (**a**) Li_2_TiS_3_, (**b**) Li_2_TiSeS_2_, and (**c**) Li_2_TiSe_2_S electrodes at different states of charge: pristine, at the end of charge (3 V), and at the end of discharge (1.6 V).

To investigate why Se-containing material cells failed upon cycling, and to get some hint of the nature of the low-potential process, we conducted ex situ structural analyses of the electrodes at different states of charge. The results of Li_2_TiS_3_, Li_2_TiSeS_2_, and Li_2_TiSe_2_S are shown in Figure 7. For both samples, we observed a reversible structural change in the diffraction patterns of the electrodes: the cubic structure became amorphous at the end of the charge, then it recrystallized to a disordered rocksalt form at the end of the discharge. Such flexible structural change in electrodes was previously reported for Li_2_TiS_3_ [19]. Differently from Li_2_TiS_3_, metallic selenium was detected in selenium substituted electrodes at the end of the charge, and disappeared at the end of the discharge (Figure 7). 

**Figure 8:**
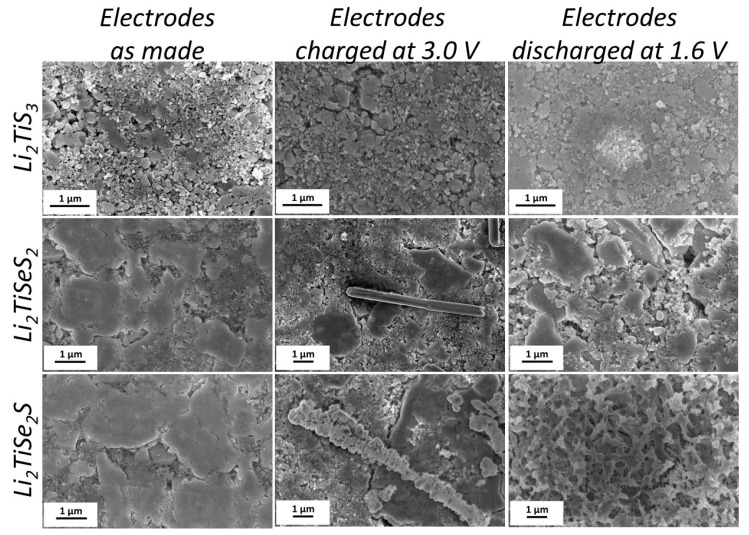
SEM images of Li_2_TiS_3_, Li_2_TiSeS_2_, and Li_2_TiSe_2_S electrodes at different states of charge: pristine, at the end of charge (3 V) and at the end of discharge (1.6 V).

**Figure 9:**
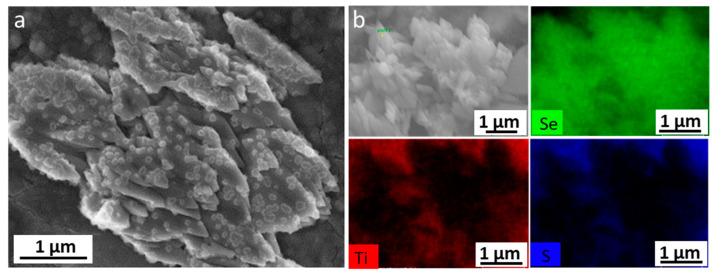
(**a**) SEM images and (**b**) EDX images of Li_2_TiSe_2_S electrodes charged to 3 V and kept for 24 h.

Moreover, the morphologies of electrodes at the different states of charge were observed with scanning electron microscopy (SEM). In Figure 8, we can see similar electrode morphologies after charge and discharge of the Li_2_TiS_3_ electrodes. In both Li_2_TiSeS_2_ and Li_2_TiSe_2_S electrodes, a columnar phase was observed at charged electrodes, whereas only one phase was observed in the discharged electrodes. Such a result is in accordance with the ex situ XRD result; metallic selenium (Se^0^) was formed at the end of the charge and no longer detectable at the discharged state. This disappearing could be due to the dissolution of the Se in the form of lithium polyselenides, which would explain the low potential process. To confirm the nature of this extra phase, a Li_2_TiSe_2_S electrode was maintained for 24 h at 3.0 V in a potentiostatic mode, to favor the hypothetical formation of Se, and then observed by microscopy. Large agglomerates were observed, which had different habitus from the starting phase (Figure 9). Their analysis by EDX confirmed that these particles were solely composed of Se.

To rule out the possible presence of Se in the pristine material, which would have stayed unnoticed due to the nano-size or to an amorphous nature, and would then follow an Ostwald ripening process or an agglomeration of particles in the electrolyte to form bigger particles [33], we performed high-resolution X-ray photoelectron spectroscopy (XPS) on the pristine powders. 

**Figure 10:**
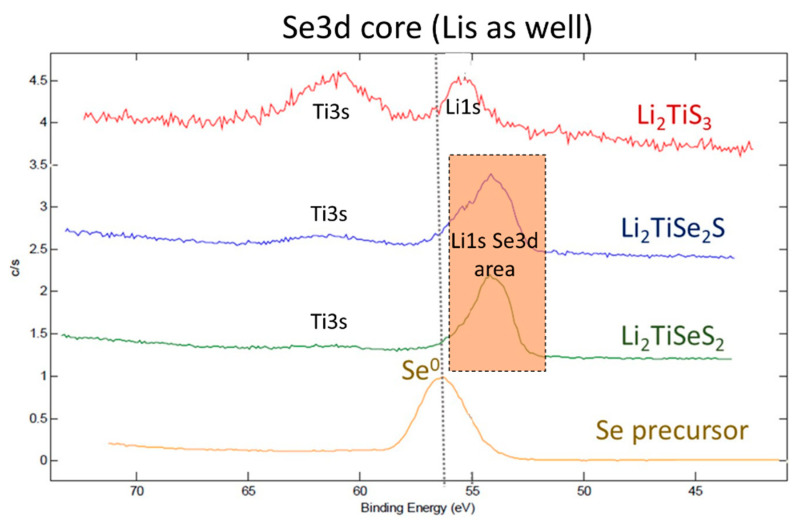
XPS spectra of powders: Li_2_TiS_3_, Li_2_TiSeS_2_, and Li_2_TiSe_2_S and selenium precursor at Se3d core.

Figure 10 shows the Se3d core peak of Li_2_TiS_3_, Li_2_TiSeS_2_, and Li_2_TiSe_2_S powders and selenium precursor. The four spectra were calibrated based on C 1s core peak at 284.8 eV.

First, it is possible to notice that the Ti3s signals in the Se-substituted samples are very weak. We suggest the hypothesis that this phenomenon is due to the Se3d cross-section, which is higher than the Ti3S one (respectively, 0.934 vs. 0.473). As seen in the figure, the binding energy of Se3d (Se^2−^) was rather lower than Se^0^, and, unfortunately, Li1s and Se3d binding energies were quite close to each other (detected between 52 eV and 55 eV, respectively). Even if the Li1s peak overlaps with that of Se3d, the cross section (sensitivity factor) of Se3d (0.821) is much higher than Li1s (0.028). Therefore, even if it is difficult to attribute the Li1S/Se3d peaks, due to possible polarization when S atoms are substituted by Se atoms (environment modification), the peak is mainly related to Se3d.

The core peak of Se^0^ was detected at 56 eV. A similar binding energy of Se^0^ was reported previously [34]. Overall, no signature of selenium precursors was detected in the XPS spectra, and, consequently, we conclude that there was no metallic selenium remaining in the powders after the synthesis.

The origin of metallic selenium seen at the end of the charge could result from the selenium redox activity; therefore, further XPS analyses on selenium-sulfide electrodes at different states of charge are required to investigate this fact. 

## 4. Conclusions

In summary, selenium-substituted samples (Li_2_TiSeS_2_ and Li_2_TiSe_2_S) were synthesized in disordered rocksalt phase, with a flexible and versatile synthesis process. Unlike Li_2_TiS_3_ cells, Li_2_TiSeS_2_ and Li_2_TiSe_2_S provided larger discharge capacities than the theoretical capacities. In cyclic voltammetry tests, Li_2_TiSeS_2_ and Li_2_TiSe_2_S showed different oxidative and reductive potentials from Li_2_TiS_3_, indicating a different redox activity. We also detected a second phenomenon that leads to extra discharge capacities. XPS and SEM and XRD ex situ studies showed that this extra capacity was coming from the activity of metallic Se that was formed during the first charge in these substituted samples and led to the formation of soluble polyselenides during the next discharge. The possible shuttle mechanism known for these species can be the origin of the low cycle life of these samples when cycled at low potential. Further structural studies are needed to elucidate the redox activities of Li_2_TiSe_x_S_3−x_.

## Figures and Tables

**Table 1:** Summary of electrochemical performances of Li_2_TiSe_x_S_3−x_ (0 ≤ x ≤ 2) materials.

Composition	Lattice Parameter a (Å)	1st Charge Capacity (mAh·g^−1^)	1st Discharge Capacity (mAh·g^−1^)	Average Charge Potential (V vs. Li^+^/Li)	Average Discharge Potential (V vs. Li^+^/Li)
Li_2_TiS_3_	5.0831	300	339	2.46	2.23
Li_2_TiSeS_2_	5.1729	179	310	2.34	2.06
Li_2_TiSe_2_S	5.2459	149	379	2.24	1.98

## Data Availability

No Supporting Information. Figures and tables are taken from the dissertation of the author, Yagmur Celasun.
